# Emergency Department and Radiological Cost of Delayed Diagnosis of Cannabinoid Hyperemesis

**DOI:** 10.1155/2019/1307345

**Published:** 2019-01-01

**Authors:** David I. Zimmer, Ross McCauley, Varun Konanki, Joseph Dynako, Nuha Zackariya, Faadil Shariff, Joseph Miller, Sophia Binz, Mark Walsh

**Affiliations:** ^1^Indiana University School of Medicine, South Bend, IN 46617, USA; ^2^Beth Israel Deaconess Medical Center, Boston, MA 02215, USA; ^3^Indiana University Bloomington, Bloomington, IN, USA; ^4^Department of Emergency Medicine, Henry Ford Hospital, Detroit, MI 48202, USA; ^5^St. Joseph Regional Medical Center, Mishawaka, IN 46545, USA

## Abstract

*Background. *Chronic cannabis use has become prevalent with decriminalization, medical prescription, and recreational legalization in numerous US states. With this increasing incidence of chronic cannabis use a new clinical syndrome has become apparent in emergency departments and hospitals across the country, termed Cannabinoid Hyperemesis (CH). CH has been described as cyclical vomiting and abdominal pain in the setting of chronic cannabis use, which is often temporarily relieved by hot showers. CH presents a diagnostic challenge to clinicians who do not have a high clinical suspicion for the syndrome and can result in high costs and resource utilization for hospitals and patients. This study investigates the expenditures associated with delayed CH evaluation and delayed diagnosis.* Methods. *This is a retrospective observational study of 17 patients diagnosed with CH at three medical centers in the United States from 2010 to 2015, consisting of two academic centers and a community hospital. Emergency department (ED) costs were calculated and analyzed for patients eventually diagnosed with CH.* Results*. For the 17 patients treated, the total cost for combined ED visits and radiologic evaluations was an average of $76,920.92 per patient. On average these patients had 17.9 ED visits before the diagnosis of CH was made.* Conclusion. *CH provides a diagnostic challenge to clinicians without a high suspicion of the syndrome and may become increasingly prevalent with current trends toward cannabis legalization. The diagnosis of CH can be made primarily through a thorough history and physical examination. Awareness of this syndrome can save institutions money, prevent inappropriate utilization of healthcare resources, and save patients from unnecessary diagnostic tests.

## 1. Introduction

In 2004, Allen et al. recognized and introduced a new clinical syndrome surrounding chronic cannabis use, coining it Cannabinoid Hyperemesis (CH) [1]. CH is characterized by cyclical vomiting and abdominal pain, both of which are temporarily relieved by hot showers [1]. The only known curative measure is cessation of cannabis use, while its reintroduction eventually leads to a recurrence of the symptoms. Simonetto et al. further clarified the criteria for diagnosis of CH to be (1) recurrent vomiting that is (2) preceded by long-term cannabis use and (3) not explained by any other major illness.

Subsequent to its description, there have been many case reports and series confirming the existence of CH [2]. In spite of Allen's first presentation in 2004, a lack of clinical recognition of this common condition persists. In fact, until recently, this condition was referred to as “rare” in the literature [3]. With steady advances in recreational cannabis legalization throughout the United States, the syndrome's representation in the literature is increasingly prevalent, but an objective cost analysis of delayed CH diagnosis has yet to be described [4–6]. Legalization of cannabis in Colorado alone has led to a twofold increase in the presentation of this syndrome [7]. In addition, the frequent recommendation that cannabis be used for the treatment of nausea will inevitably result in a further increased incidence of this syndrome. CH has been underappreciated in ED settings, leading to increased expenditures and unnecessary radiation exposure when diagnosis is delayed [8].

The association of chronic cannabinoid use and cyclical vomiting remains a paradox that has been theorized to be due to several mechanisms, including fat-soluble prolongation of cannabinoid half-life, delayed gastric emptying, and thermoregulatory disturbances via the limbic system [2, 9]. An intriguing and consistent component of these cases, which is necessary for the diagnosis, is compulsive bathing and showering in hot water, which provides transient relief of symptoms. It has been proposed that chronic stimulation of CB1 vascular receptors in the gut leads to their post synaptic upregulation in the splanchnic circulation. This increased upregulation, similar to the regulation found in gut vascular receptors during sepsis and later stages of cirrhosis, can lead to hyperperfusion of the viscera, which might cause abdominal pain. It is possible that the upregulated CB1 receptor-mediated vasodilation of the abdominal viscera contributes to the symptoms caused by chronic cannabinoid use. The transient relief of symptoms following prolonged warm bathing or showering may be related to a redistribution of blood flow from the gut to the skin. This relief of symptoms caused by the proposed redistribution of blood flow from the splanchnic circulation to the cutaneous circulation has been described as the “Cutaneous Steal Syndrome” [9].

CH has also been described in patients after the use of synthetic cannabis-like substances [10]. It can therefore be anticipated that the combination of the previously documented increase in incidence of CH, associated with legalization of cannabis, the recommendation of medicinal cannabis, and the aforementioned proliferation of synthetic cannabis products, will lead to an overall dramatic increase in the presentation of these patients to the ED [7, 8, 10].

Radiologic costs have recently been shown to be the greatest expense in ED visits for abdominal pain with a low rate of true positive findings [11]. As CH patients have multiple ED admissions and many radiologic tests prior to diagnosis, the costs of undiagnosed CH are likely high and even harmful to patients. Previously, there have only been preliminary analyses of the cost of CH in ED settings [12]. In order to quantify the cost associated with delayed CH diagnosis and work-up, we have reviewed a convenience sample of patients presenting with a final diagnosis of CH and calculated the cost for the ED and radiologic diagnostic evaluation prior to the final diagnosis.

## 2. Methods

This was a retrospective observational study of 17 patients diagnosed with CH at three medical centers in the United States from 2010 to 2015, consisting of two academic centers and a community hospital. Diagnosis of CH was made clinically when patients met all four of the following criteria: (1) intractable abdominal pain, nausea, and/or vomiting, (2) relief of pain and nausea (with or without vomiting) by hot baths or showers, (3) history of daily cannabis use, and (4) absence of another obvious cause of symptoms. Charts were then reviewed for patients who were diagnosed with CH at one of the participating institutions from 2010 to 2015. In order to evaluate the cost of delayed CH diagnosis, patients with four or more admissions to the ED, who met the CH diagnosis criteria at each of these visits, from 2010 to 2015 were enrolled in the study. Patients were cross-referenced to ensure that they were not counted as participants at the other study hospitals. Total ED costs were calculated based on patient charges for the ED visits, and various radiographic testing including X-rays, CT scans, and ultrasounds; the median cost between the three institutions of each measured metric was used. ED costs were defined as the costs incurred while the patients were in the ED, and hospital costs were excluded from the analysis.

## 3. Results

For the 17 patients seen, the total cost for combined ED visits and radiologic evaluations was an average of $76,920.92 per patient (Table 1).

On average these patients had 17.9 ED visits before the diagnosis of CH was made, with a range from 5 to 38 visits per person and incurred per-patient total radiologic costs ranging from $17,133 to $210,010 (Supplemental Table 1). Until the diagnosis of CH was made, patients were exposed to an average of 5.94 radiographs, 4.94 CT scans, and 2.41 ultrasounds (Supplemental Table 1). In addition, among the 17 patients included, there were 58 total hospital admissions, 3 surgical interventions (an appendectomy and two cholecystectomies), 8 colonoscopies, and 17 esophagoduodenoscopies (EGDs).

## 4. Discussion

CH has become a much more commonly recognized disease process as cannabis use has become much more transparent with the decriminalization and legalization of cannabis in numerous US states. An increase in patients presenting with CH, coupled with its underrecognition in the ED, is costly and leads to wasted resources and unnecessary radiation exposure, evidenced by the average CH evaluation cost prior to diagnosis of $76,290.92 per patient in these three hospitals under study.

We elected to present the radiologic data as it pertains to CH because of its objective quantitation among institutions; however the actual costs of misdiagnosis of CH are far greater. As indicated by the results above, there were a very large number of total inpatient hospitalizations, EGDs, and colonoscopies and even three surgical interventions. It is important to note that these hospitalizations, surgeries, and interventions revealed no pathology other than two patients diagnosed with unspecific gastritis. These data are not included in the costs of delayed diagnosis of CH but are mentioned to convey the further economic burden and exposure of unnecessary interventions in patients eventually diagnosed with CH.

Furthermore, radiological burden on patients undergoing CT scans and radiograph studies is not insignificant. Hall et al. have showed that excess relative risk is statistically significant at only 35 mSv of radiation exposure [13]. A typical dose from a diagnostic CT scan is 10–20 MsV, exposing our cohort to 49.4–98.8 mSv during their evaluation. It should be noted that individual risk has been shown to be small and difficult to quantify and current epidemiologic studies of the subject have yet to be published. For a population perspective, however, it is estimated that 1.5-2.0% of all cancers in the United States may be attributed to radiation from CT studies [14].

Being a diagnosis based largely on history and physical examination, physicians cognizant of CH who maintain a high degree of suspicion for presentation in their patient populations, can enable considerable savings and prevent unnecessary testing. Informing ED clinicians of CH is therefore universally beneficial, especially given its infancy in mainstream medical literature as of 2018 [3].

Limitations of this study begin with a small sample size as it was based on chart review from two academic medical centers and a community hospital and was comprised of only 17 patients with a final diagnosis of CH. Second, only patients with a diagnosis of CH who had at least four visits to the ED meeting CH diagnosis criteria were included in the study, which does not account for any patients who may have received a CH diagnosis before the fourth visit. This methodology could potentially overestimate average cost incurred by patients before CH diagnosis but was chosen to attempt to evaluate the cost of delayed CH diagnosis. However, charges for laboratory tests, medications, IVs, and inpatient costs were excluded from the analysis which would have increased the cost incurred per patient. Finally, the cost incurred per patient was calculated based only on what was billed to each patient, rather than what was paid by the patient and their insurance company, which does not account for differences in insurance reimbursement.

## 5. Conclusion

CH provides a diagnostic challenge to clinicians without a high suspicion of the syndrome. The diagnosis of CH can be made primarily through a thorough history and physical examination. CH patients often have multiple ED admissions and many radiologic tests prior to diagnosis. The costs of undiagnosed CH are high and even potentially harmful to patients [15]. Awareness of this syndrome and high clinical suspicion can save institutions money, prevent inappropriate utilization of healthcare resources, and save patients from unnecessary diagnostic tests.

## Figures and Tables

**Table 1:** Costs of delayed diagnosis of CH in ED hospital visits (ED: emergency department; AAS: acute abdominal series; CT: computed tomography; US: ultrasound).

Number of ED Admissions Per Patient	17.9
Average Total ED Charge	$ 36,188.52
Average Number of X-Rays	0.9
Average Total Cost of X-Rays	$756.78
Average Number of AAS	5
Average Total Cost of AAS	$4,189.50
Average Number of CTs	4.9
Average Total Cost of CTs	$31,092.23
Average Number of US	2.4
Average Total Cost of US	$4,063.89
Average Total Cost of All Imaging	$40,102.40
Average Total Cost Incurred Per Patient	$76,290.92

*∗*X-ray is constituted by a single radiograph. An acute abdominal series (AAS) is a radiological exam consisting of a series of radiographs that includes an erect kidney ureter and bladder (KUB) projection, a recumbent KUB projection, and a left lateral decubitus image of the abdomen.

## Data Availability

The raw data used to support the findings of this study are included within the supplementary information file.

## Supplementary Materials

Supplementary Materials Raw data for total number of visits per patient to the ER is summarized in Supplemental Table 1.
