# Supplementary material for: Emergency Department and Radiological Cost of Delayed Diagnosis of Cannabinoid Hyperemesis
Source: J Addict. 2019 Jan 1;2019:1307345. doi: 10.1155/2019/1307345 (PMC6339733; doi:10.1155/2019/1307345)
Supplement: Supplementary Materials — Raw data for total number of visits per patient to the ER is summarized in Supplemental Table 1. [file 1307345.f1.docx]

Supplemental Table 1: Raw Data for total number of visits per patient to the ER, total number of imaging studies, and associated cost for visits and imaging (ED-emergency department, PA-posterior-anterior, Lat-lateral, W-with, WO-with out, AAS-abdomen acute series, CT -computed tomography, U/S-ultrasound).

| Patient | # ED admissions | Total Cost of All ED Visits ($2023.7/visit) | X Rays PA and Lat ($735.15) | DX Abdomen Complete W-Chest ($857.69) | X Rays Total | Abdomen Acute Series | AAS Total Charges ($837.90) | CT pelvic with contrast ($3363.99) | CT ABD with contrast ($3651.11) | CT ABD/P with contrast $7052 | Total CT Charges | U/S Pelvic | Pelvic U/S Charges ($1481) | U/S Abdominal | Abdominal U/S Charges ($1742.41) |
| --- | --- | --- | --- | --- | --- | --- | --- | --- | --- | --- | --- | --- | --- | --- | --- |
| 1 | 6 | $12,142.20 | 1 | 0 | $735.15 | 0 | 0 | 1 | 1 | 2 | $21,119.1 | 0 | 0 | 0 | 0 |
| 2 | 5 | $10,118.50 | 0 | 0 | 0 | 0 | 0 | 1 | 1 | 0 | $7,015.1 | 0 | 0 | 0 | 0 |
| 3 | 28 | $56,663.60 | 0 | 2 | $1,715.38 | 0 | 0 | 2 | 2 | 4 | $42,238.2 | 1 | $1,481 | 2 | $3,484.82 |
| 4 | 25 | $50,592.50 | 0 | 1 | $857.69 | 2 | $1,675.80 | 2 | 2 | 1 | $21,082.2 | 0 | 0 | 0 | 0 |
| 5 | 22 | $44,521.40 | 0 | 1 | $857.69 | 0 | 0 | 1 | 1 | 0 | $7,015.1 | 0 | 0 | 0 | 0 |
| 6 | 32 | $64,758.40 | 2 | 3 | $4,043.37 | 0 | 0 | 1 | 1 | 1 | $14,067.1 | 0 | 0 | 1 | $1,742.41 |
| 7 | 5 | $10,118.50 | 0 | 0 | 0 | 0 | 0 | 1 | 1 | 0 | $7,015.1 | 0 | 0 | 4 | $6,969.64 |
| 8 | 20 | $40,474 | 4 | 2 | $4,655.98 | 2 | $1,675.80 | 0 | 0 | 2 | $14,104 | 0 | 0 | 0 | 0 |
| 9 | 15 | $30,355.50 | 0 | 0 | 0 | 8 | $6,703.20 | 0 | 0 | 2 | $14,104 | 0 | 0 | 3 | $5,227.23 |
| 10 | 14 | $28,331.80 | 0 | 0 | 0 | 8 | $6,703.20 | 0 | 0 | 8 | $56,416 | 0 | 0 | 0 | 0 |
| 11 | 8 | $16,189.60 | 0 | 0 | 0 | 5 | $4,189.50 | 0 | 0 | 4 | $28,208 | 2 | $2,962 | 3 | $5,227.23 |
| 12 | 14 | $28,331.80 | 0 | 0 | 0 | 14 | $11,730.60 | 0 | 0 | 4 | $28,208 | 0 | 0 | 5 | $8,712.05 |
| 13 | 23 | $46,545.10 | 0 | 0 | 0 | 7 | $5,865.30 | 0 | 0 | 5 | $35,260 | 0 | 0 | 1 | $1,742.41 |
| 14 | 12 | $24,284.40 | 0 | 0 | 0 | 7 | $5,865.30 | 0 | 0 | 3 | $21,156 | 1 | $1,481 | 0 | 0 |
| 15 | 11 | $22,260.70 | 0 | 0 | 0 | 5 | $4,189.50 | 0 | 0 | 4 | $28,208 | 0 | 0 | 0 | 0 |
| 16 | 26 | $52,616.20 | 0 | 0 | 0 | 13 | $10,892.70 | 0 | 0 | 12 | 84,624 | 0 | 0 | 0 | 0 |
| 17 | 38 | $76,900.60 | 0 | 0 | 0 | 14 | $11,730.60 | 0 | 0 | 14 | $98,728 | 5 | $7,405 | 13 | $22,651.33 |
|  |  |  |  |  |  |  |  |  |  |  |  |  |  |  |  |
|  |  |  |  |  |  |  |  |  |  |  |  |  |  | Total | $1,296,945.58 |
|  |  |  |  |  |  |  |  |  |  |  |  |  |  | average | $76,290.92 |

*X-Ray is constituted by a single radiograph. An acute abdominal series (AAS) is a radiological exam consisting of a series of radiographs that includes an erect kidney ureter, and bladder (KUB) projection, a recumbent KUB projection, and a left lateral decubitus image of the abdomen. Abdomen complete with chest consists of an AAS with the addition of an upright posterior- anterior (PA) radiograph of the chest.

**Costs reflect figures billed to patients for services received during emergency department visits
